# Osteochondroma of talus: A case report from Nepal

**DOI:** 10.1002/ccr3.5867

**Published:** 2022-05-12

**Authors:** Ajay Shah, Shirish Adhikari, Sangam Shah, Prawesh Singh Bhandari, Suresh Uprety

**Affiliations:** ^1^ Department of Orthopedics Maharajgunj Medical Campus Tribhuvan University Maharajgunj Nepal; ^2^ Maharajgunj Medical Campus Institute of Medicine Tribhuvan University Maharajgunj Nepal

**Keywords:** Nepal, osteochondroma, talus

## Abstract

Osteochondroma, the most frequent benign bone tumor, develops in the metaphysis of long bones including the proximal humerus, tibia, and distal femur. The involvement of talus is found only in a few patients. Here, we present a case of osteochondroma of the talus in a 52‐year‐old woman who presented with ankle pain and edema.

## Abstract 2

Talus may be affected in the osteochondromas. Surgical excision is a successful technique of treatment for symptomatic osteochondromas with low recurrence, despite the fact that most osteochondromas are treated conservatively.

## 1 INTRODUCTION

An osteochondroma, also known as exostosis, is a benign bone tumor that develops in the metaphysis of long bones, most commonly the distal femur, proximal tibia, and proximal humerus. It is made up of a bony protrusion that is covered by a cartilage cap.^1^ It is the most frequent benign bone tumor. It is very frequent in kids and young adults.^2^ It is normally asymptomatic and discovered by chance during a routine radiography scan, but it can become painful if it impinges on nearby tissues or joints.^3^ The most frequent benign bone tumor, osteochondroma, accounts for 20%–50% of benign bone tumors and 9% of all bone tumors.^4^ The occurrence of an osteochondroma in the talus is quite uncommon. Only a few cases of isolated talar osteochondroma have been reported. Here, we report a case of a 52‐year‐old female patient with solitary benign osteochondromas of the talus presenting with ankle pain and swelling.

## 2 CASE DESCRIPTION

**FIGURE 1:**
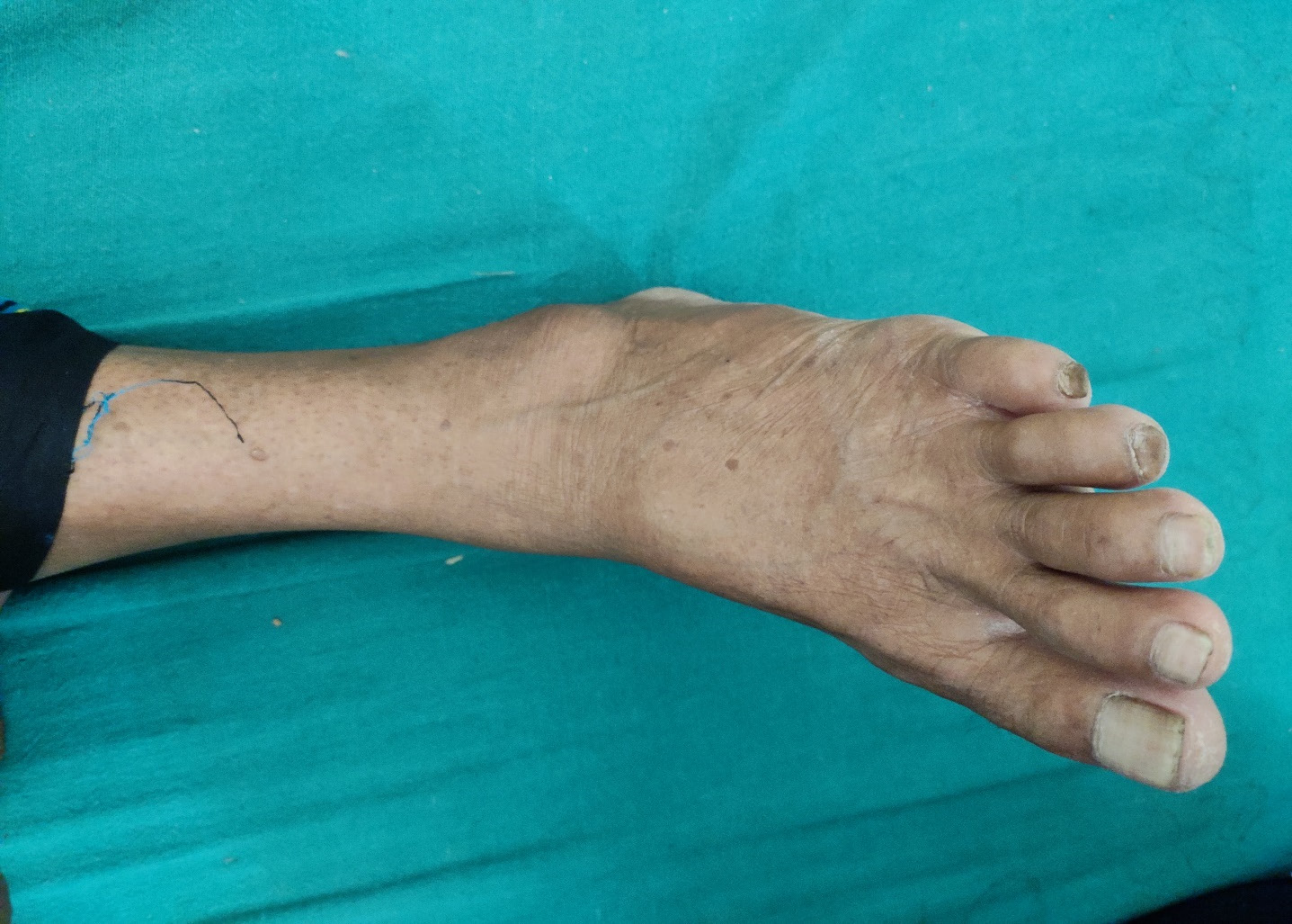
Clinical photograph of left ankle showing swelling below lateral malleolus

A 52‐year‐old female patient presented to us with complaints of pain in her left ankle for one year. The pain was insidious in onset, dull aching, that aggravated on walking and standing from a sitting position. The patient also complained of swelling around the ankle [Figure 1]. The swelling started out little and progressively grew to the size of a marble. Edema of the foot occurred on occasion, which increased with walking and decreased with rest and limb elevation. A single bony hard swelling was palpable right below the lateral malleolus on inspection. The ankle's range of motion was normal, but there was painful pronation. The arteries of the tibialis posterior, dorsalis pedis, and tibialis anterior were all palpable. Over the lateral area of the foot and ankle, there was no motor weakness or altered sensation.

**FIGURE 2:**
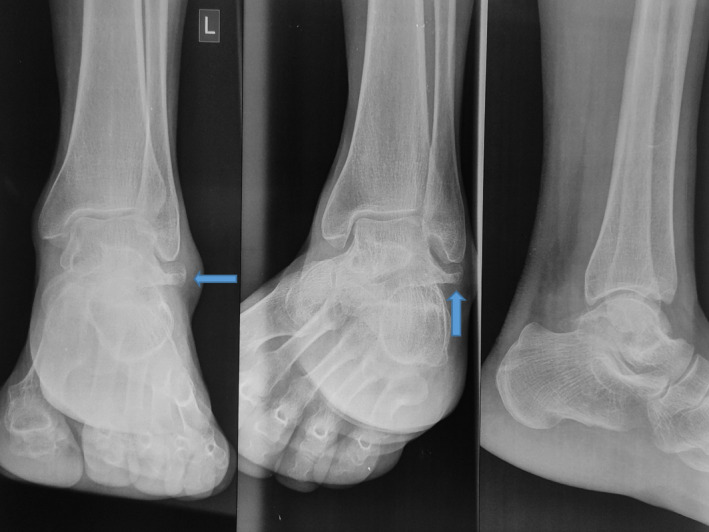
Radiographs of ankle showing bony outgrowth from lateral aspect of talus just below the lateral malleolus (arrow)

The results of routine laboratory tests were within normal limits. Radiographs showed solitary bony outgrowth from the lateral aspect of the talar body [Figure 2].

**FIGURE 3:**
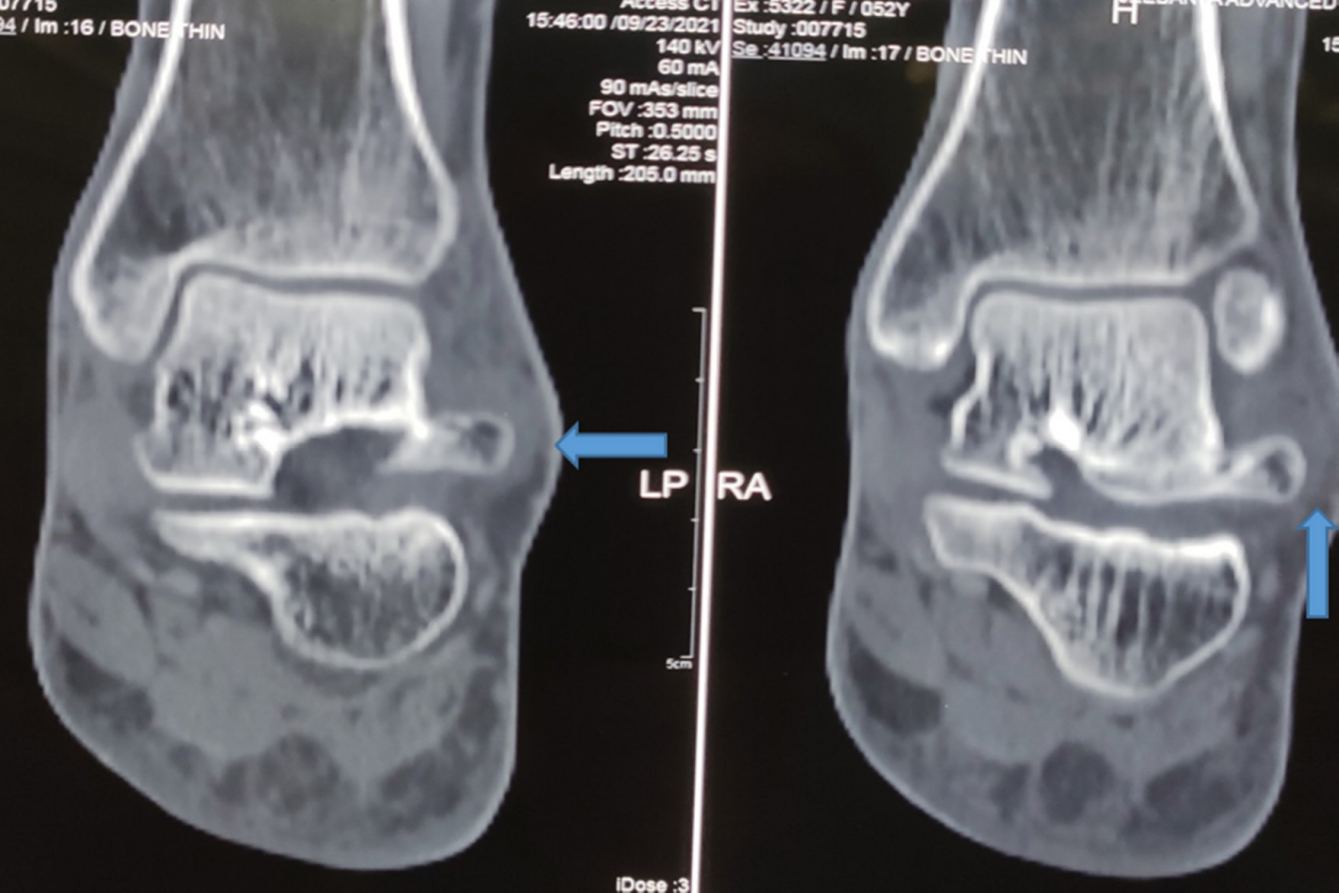
Coronal CT image of left ankle showing a bony outgrowth from the lateral aspect of body talus (arrow)

Computed tomography (CT) scan was done which showed approximately 11.1 mm (long) and 9.4 mm (width) cortically based bony outgrowth noted from the lateral aspect of the body part of the talus with minimal thickening of overlying soft tissue [Figure 3].

**FIGURE 4:**
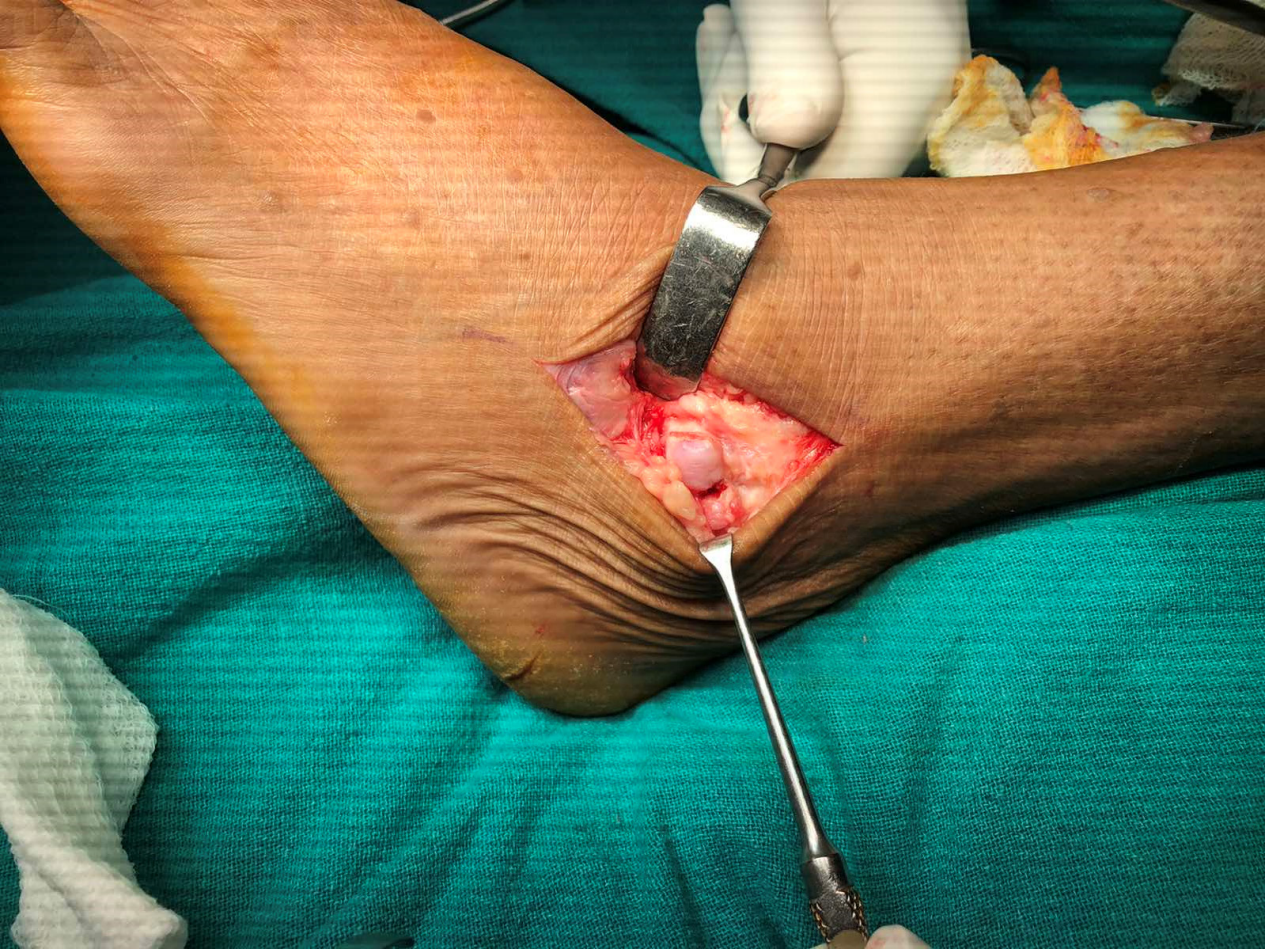
Intra‐operative photograph showing cartilage‐capped bony mass

**FIGURE 5:**
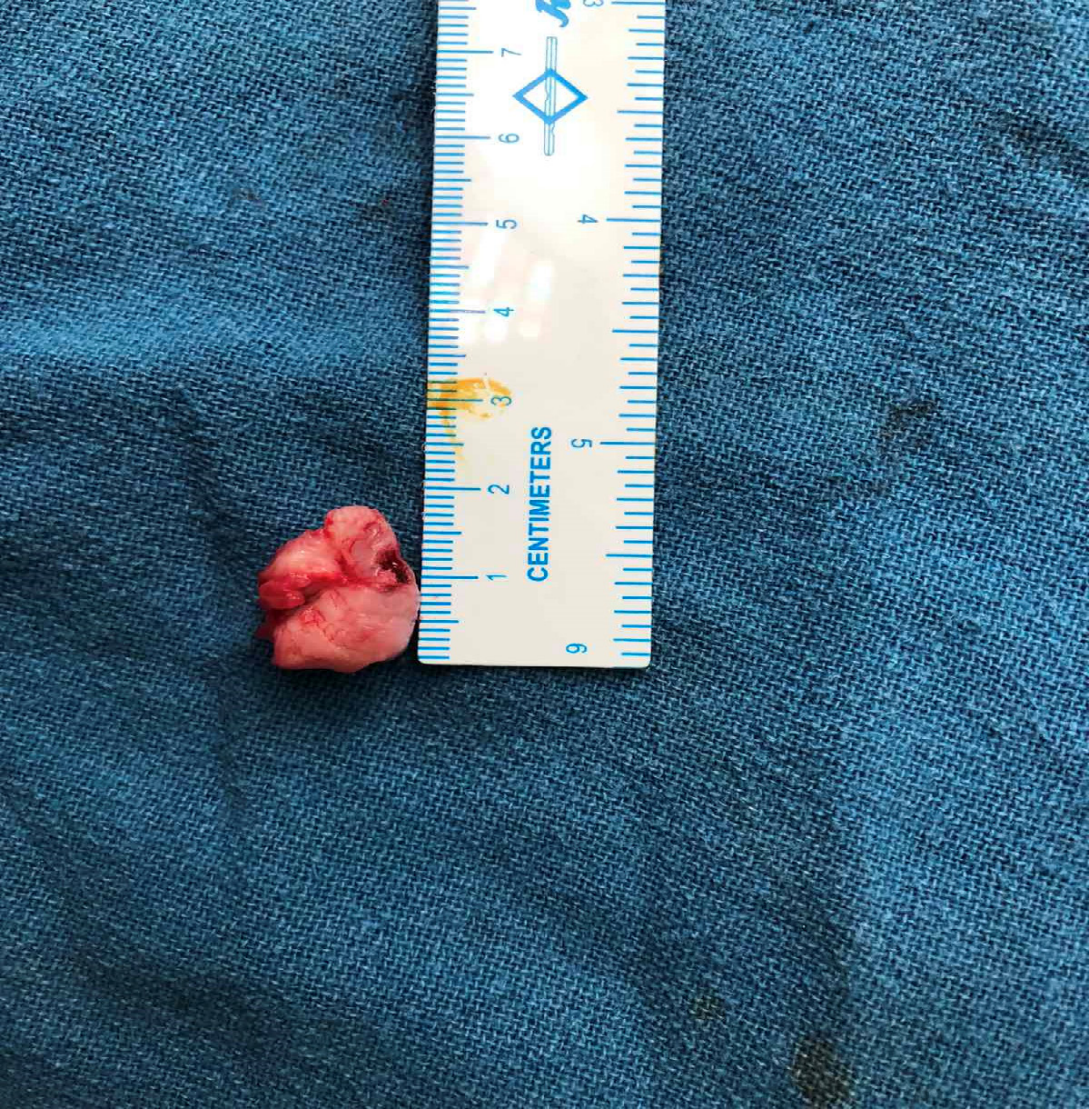
Macroscopic appearance of excised bony outgrowth

A radiological diagnosis of osteochondroma was made, and she was planned for excision of exostosis. It was decided to approach the talus from the anterolateral side due to exostosis on the anterior and lateral sides of the talar body. On the anterolateral part of the ankle, an 8 cm curvilinear incision was created. On exposure, a cartilage‐capped bone mass was discovered [Figure 4], which was excised in its entirety with the help of an osteotome. [Figure 5] The wound was closed with interrupted non‐absorbable sutures.

**FIGURE 6:**
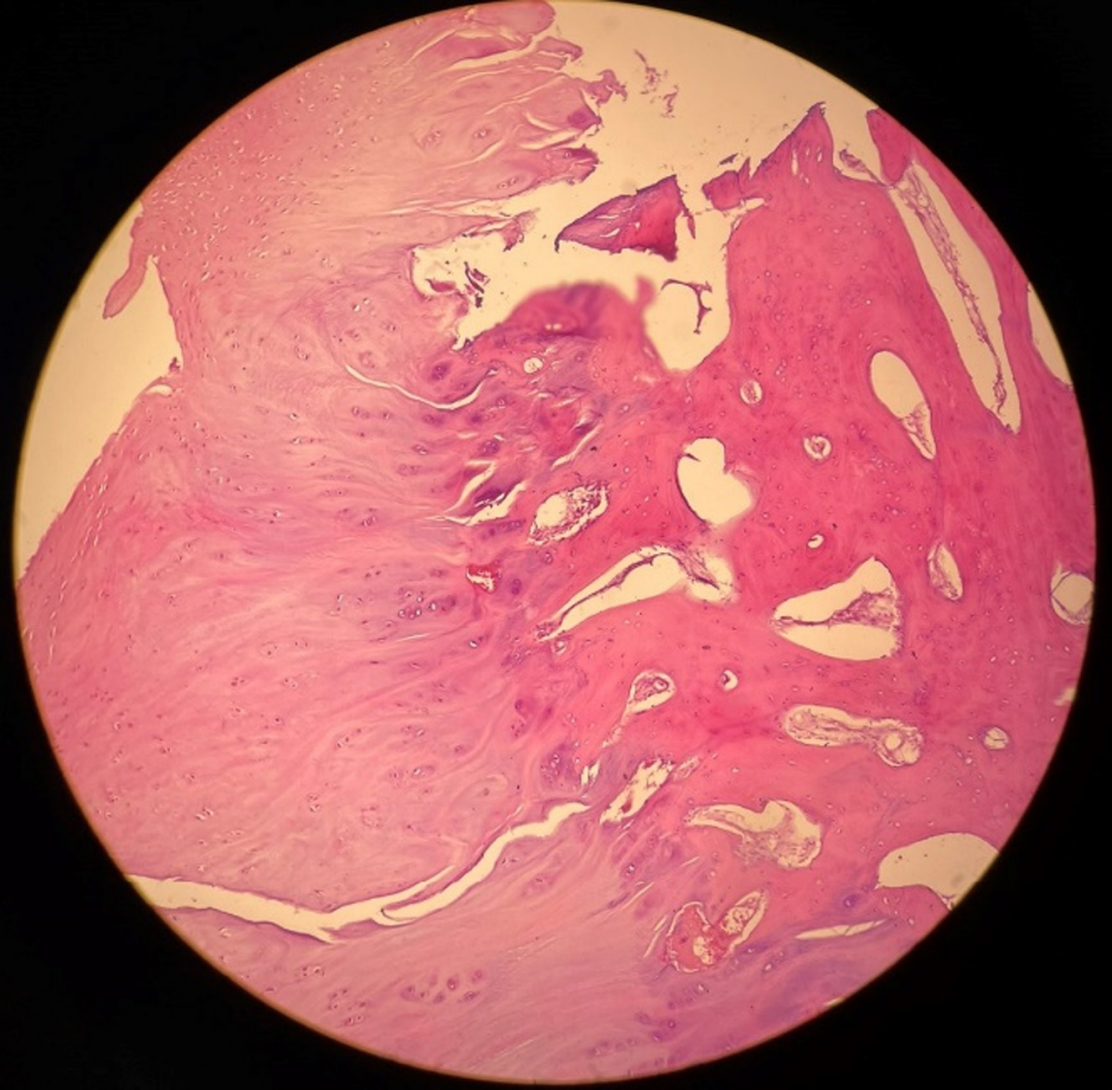
Histopathological examination revealed primary trabecular bone covered with cartilaginous cap consisting of hyaline cartilage with well‐defined perichondrium around the cartilage cap

Histopathological examination revealed primary trabecular bone covered with a cartilaginous cap consisting of hyaline cartilage with well‐defined perichondrium around the cartilage cap. Linear clusters of active chondrocytes were seen having thin cartilaginous cap covering the lesion suggestive of osteochondroma [Figure 6].

**FIGURE 7:**
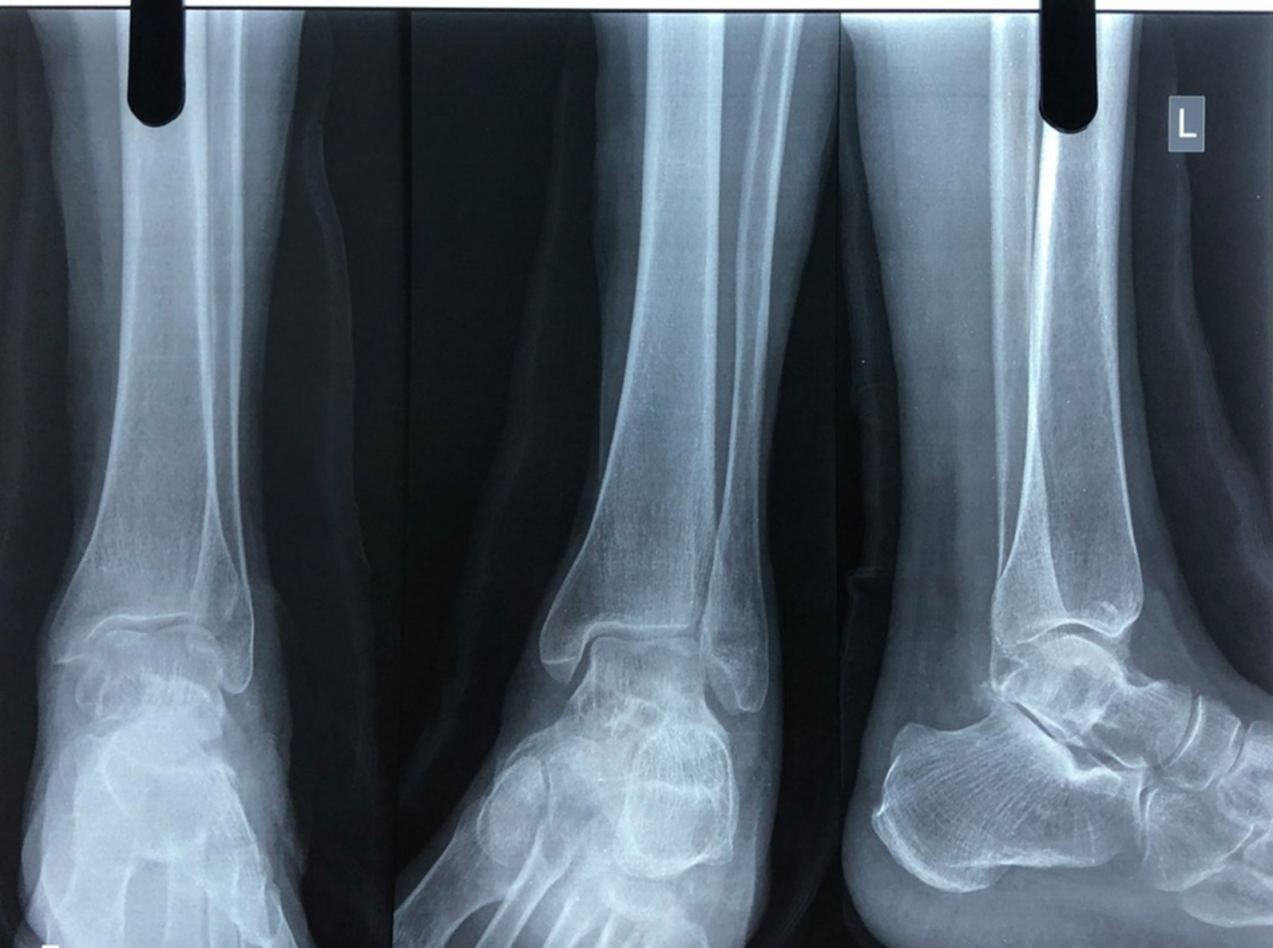
Postoperative radiographs showing complete excision of mass

The post‐operative time went smoothly. The mass was completely removed on post‐operative radiography. [Figure 7] The patient's symptoms improved after surgery. On the third post‐operative day, the patient was allowed to begin full weight‐bearing. On follow‐up after three weeks, she has no fresh issues and there was no recurrence.

## 3 DISCUSSION

The most common benign bone tumor is an osteochondroma, which affects the proximal humerus, tibia, and distal femur.^5^ Osteochondromas can develop in any bone that is cartilage‐covered. The metaphyseal area of the long bones is the most prevalent location.^1^ It accounts for around 36%–41% of all benign bone tumors.^4^ Only 15 osteochondromas were found in the tarsal region of a series of 783 osteochondromas, 10 of which were in the calcaneus.^6^ It occurs seldom in foot bones and even less frequently in the talus.^7^

Fuselier et al. described the first osteochondroma of the talus in 1984.^8^ Chioros et al. described an unusual osteochondroma in a 34‐year‐old male patient that arose from the posterior side of the talus in 1987.^7^ An osteochondroma on the dorsum of the talus was documented by Erler et al.^9^

The majority of osteochondromas are asymptomatic. However, as in our patient who reported ankle discomfort and swelling with restricted ankle pronation, osteochondroma of the talus might present with pain in the ankle or foot, as a painless ankle mass, decreased range of ankle motion, and other symptoms.^10^ Simple removal is the surgical treatment for osteochondromas; however, Boya et al.^11^ stressed the need for extra‐periosteal full resection of the cartilaginous cap to prevent a recurrence. Excision of the bony outgrowths from the lateral aspect of the talus was performed in our case.

Because the histology and radiographic features are so similar, osteochondroma of the talus must be distinguished from Trevor's disease.^12^, ^13^ The latter has a cartilage border and a normal epiphysis before complete ossification.^12^ An osteochondroma in the talus is quite uncommon, according to previous research. Even infrequent are osteochondromas on the anterior and anteromedial sides of the talus, which might impede ankle movement as the primary clinical complaint.

## 4 CONCLUSION

Osteochondroma should be considered a differential diagnosis for any mass around the ankle, whether it is painful or not. The most effective treatment for symptomatic osteochondroma is surgical resection with complete tumor excision.

## AUTHOR CONTRIBUTIONS

AS wrote the original manuscript, reviewed, and edited the original manuscript. SS, SA, PSB, and SA reviewed the original manuscript and were in charge of the case.

## CONFLICT OF INTEREST

None.

## ETHICAL APPROVAL

None.

## CONSENT

Written informed consent was taken from the patient for publication of the report.

## Data Availability

All the required data are available in the manuscript itself.
